# A National Survey of Provider Practice Patterns for Evaluation and Management of Patients With Urinary Stone Disease

**DOI:** 10.1016/j.xkme.2026.101520

**Published:** 2026-09-09

**Authors:** Yunseo Jo, Calyani Ganesan, Lindsey Keo, Glenn M. Chertow, Simon John Christoph Soerensen, Maria Montez-Rath, John T. Leppert, Alan C. Pao

**Affiliations:** 1 Division of Nephrology, Department of Medicine, Stanford University School of Medicine, Stanford, CA; 2 Veterans Affairs Palo Alto Health Care System, Palo Alto, CA; 3 Department of Urology, Stanford University School of Medicine, Stanford, CA

**Keywords:** Urinary stone disease, kidney stones, nephrolithiasis, 24-hour urine testing, metabolic evaluation, practice patterns, survey

## Abstract

**Rationale & Objectives:**

Urinary stone disease (USD) is increasing in prevalence and contributes to significant morbidity and health care costs. Use of secondary prevention measures remains low. We aimed to characterize provider practice patterns, barriers, and attitudes toward metabolic evaluation, treatment, and radiologic surveillance of USD.

**Study Design:**

A national cross-sectional survey from 2024 to 2025.

**Setting and Participants:**

Physicians, nurse practitioners, and physician assistants who care for patients with USD.

**Exposure:**

American Urological Association guidelines for medical management of USD

**Outcomes:**

Implementation of 24-hour urine testing, dietary and pharmacologic interventions, imaging practices, and perceived barriers to secondary prevention

**Analytical Approach:**

Responses were summarized descriptively and stratified by specialty.

**Results:**

Ninety-three respondents completed the survey (88% physicians, 46% urology, and 35% nephrology); the remaining were advanced practice providers. Overall, 60% reported ordering 24-hour urine testing in all or more than half of their patients, though frequency differed by specialty (72% of nephrologists compared with 63% of urologists). Most (93%) agreed that 24-hour urine testing is necessary and felt confident in interpreting results. The leading barrier was the perceived burden for patients to complete urine collections (35%). Among respondents who obtained 24-hour urine testing, 77% interpreted results for dietary counseling and 91% for medication selection. Imaging strategies also varied: 32% of nephrologists, when compared with 11% of urologists, rely on computed tomography alone to survey stone burden. Most providers who did not practice surveillance imaging cited concerns about radiation exposure (25%) and pursued imaging only when their patients developed symptoms (80%).

**Limitations:**

Survey design was cross-sectional; most respondents were affiliated with an academic institution.

**Conclusions:**

Most providers caring for patients with USD use 24-hour urine testing to inform treatment strategies, but they may not consistently order 24-hour urine tests due to the perception that urine collections are burdensome for patients. Twenty-four-hour urine testing, prescription of stone medications, and surveillance imaging vary by specialty.

## Plain-Language Summary

Urinary stones are common and often recur. It is unclear how health care providers manage patients with urinary stone disease and what barriers impede adherence to guidelines. We conducted a national survey of health care providers across the United States and found that more than 90% of providers believe that 24-hour urine testing is necessary for guiding decisions for dietary and pharmacological treatment. However, up to 35% of providers do not always order 24-hour urine testing because they perceive it to be too burdensome for patients to collect. We also found differences in how nephrologists, urologists, and generalists approach treatment of urinary risk factors and stone surveillance through radiologic imaging. Our findings highlight opportunities to improve the implementation of stone prevention strategies and standardization of care.

Urinary stone disease (USD) is common and recurrent, causing significant patient morbidity and health care utilization in the United States.^1^ The American Urological Association and the European Association of Urology have issued guidelines on the medical management of USD,^2^^,^^3^ which include recommendations for 24-hour urine testing, dietary modification, prescription of stone-related medications, and surveillance imaging. Many of these guidelines are supported by evidence that has been deemed to be moderate strength (ie, Grade B), whereas some are informed more by expert consensus (Table S1). For example, recommendations such as increasing fluid intake to achieve adequate urine volume, limiting dietary sodium in patients with hypercalciuria, and prescribing thiazide diuretics for recurrent calcium stones with hypercalciuria are supported by Grade B evidence. Similarly, potassium citrate for hypocitraturia and allopurinol for hyperuricosuria are supported by Grade B evidence. In contrast, other guidelines such as restricting dietary oxalate, increasing fruit and vegetable intake, prescribing alkali supplements for uric acid stones, and tracking stone burden with radiological imaging are largely informed by expert opinion (Table S1).

Given the varying strength of evidence supporting guidelines, the American Urological Association acknowledges the need for individualized clinical decision-making, which may lead to variation in practice. It may thus not be surprising that the prevalence of guideline-concordant care remains low.^4^, ^5^, ^6^, ^7^, ^8^, ^9^, ^10^ At the same time, some measures, such as diet and pharmacological intervention guided by 24-hour urine testing, remain cornerstones of stone prevention. Better understanding of provider sentiments, perceived barriers, and practice patterns regarding stone prevention could help improve guideline adherence and quality of care.

We conducted a national survey of physicians and advanced practice providers who care for patients with USD. The survey aimed to characterize practice patterns, including metabolic evaluation, specifically 24-hour urine testing, diet recommendations, and medication prescriptions, and imaging studies for stone surveillance. Our overall goal was to identify current barriers to implementing guideline concordant care for USD and to better understand how to increase utilization of guidelines among different primary care and specialty providers who treat patients with USD.

## Methods

### Survey Design and Data Source

The Stanford University institutional review board (Protocol ID# 57658) deemed this study to be exempt because survey data from health care providers was kept anonymous. The requirement for informed consent was waived. We collected data through an online survey from 23 October 2024 to 13 August 2025. We designed the survey with input from nephrologists and urologists who routinely care for patients with USD (Table S2). Quick response codes linked to the online survey were distributed in person at national conferences, including the American Society of Nephrology, Research on Calculus Kinetics, and the National Kidney Foundation meetings. The quick response codes were also shared during national presentations on stone prevention measures delivered both in person and virtually through the VA ECHO program. Finally, a survey link was distributed via a national e-mail list of primary care providers and urologists within the Veterans Health Administration.

### Study Population

Respondents were eligible to participate if they provide medical care for patients with USD within the United States. Respondents included providers affiliated with the Veterans Health Administration.

### Study Content

We obtained information on provider type (physician, physician assistant, or nurse practitioner), provider specialty (nephrology, urology, or other), geographic location of practice within the United States, and monthly number of patients with USD cared for by the provider. Respondents were asked about the frequency with which they order and use 24-hour urine testing for counseling, dietary recommendations, and prescription of medications in response to specific urinary abnormalities. Respondents were also asked whether they routinely use stone surveillance imaging and, if so, the type of imaging. Finally, respondents were asked about perceived barriers to implementing 24-hour urine testing and strategies to improve utilization.

### Statistical Analyses

Study measures and outcomes were summarized for all providers and categorized by specialty (nephrology, urology, and other) in relevant instances. Frequency counts and percentages were reported for categorical variables. We used χ^2^ tests of independence to compare prescriber patterns between different provider clinics. A two-sided *P* value < 0.05 was considered statistically significant.

## Results

Ninety-three respondents completed the survey in the United States, with most respondents practicing in the regions of Wisconsin/Illinois (11 respondents), New York (7), Montana/Wyoming/Utah/Colorado (7), and northern California/western Nevada (7) (Fig 1). The majority were physicians (88.2%), followed by nurse practitioners (6.5%) and physician assistants (5.4%) (Table 1). Most respondents specialized in urology (46.2%) or nephrology (34.4%), whereas 19.4% of respondents reported belonging to general disciplines (other). More than half (53.8%) reported seeing over 50 patients with USD annually, whereas 22.6% cared for between 20 and 50 patients with USD, and 23.7% cared for fewer than 20 patients with USD annually. Most respondents (61.3%) had been practicing for more than 10 years, and most (88.2%) were affiliated with an academic institution (Table 1).

**Figure 1:**
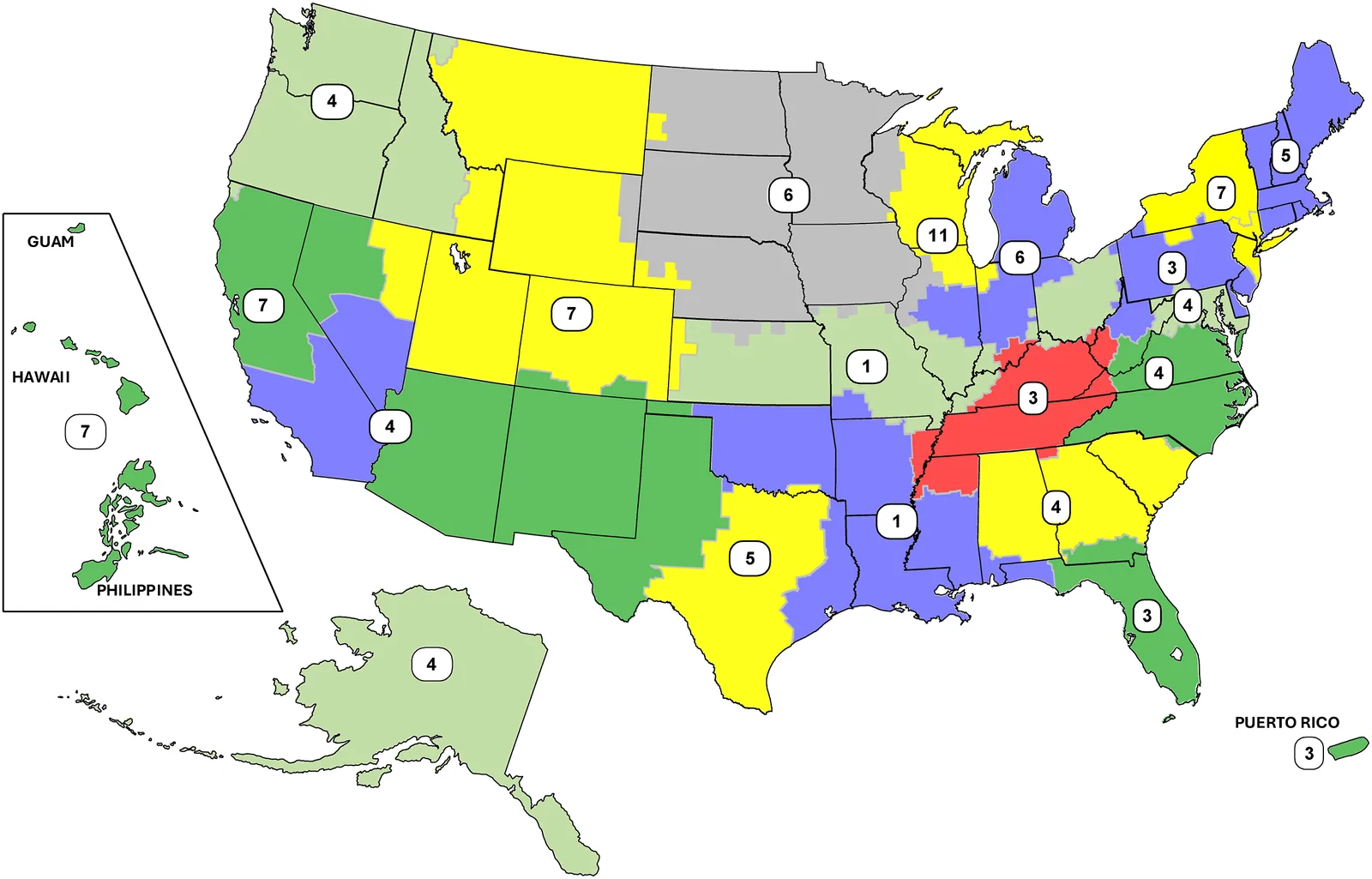
Geographical distribution of survey respondents. We used this national map to determine the geographical distribution of survey respondents. Numbers in gray circles represent the number of respondents from each region of the United States. The regions correspond to the veterans integrated service network, although survey respondents were not necessarily working in the Veterans Health Administration.

**Table 1:** Survey Respondent Characteristics

Characteristic (n,%)	Respondents (N = 93)
Provider type	
Physician	82 (88.2%)
Nurse practitioner	6 (6.5%)
Physician assistant	5 (5.4%)
Specialty	
Urology	43 (46.2%)
Nephrology	33 (35.4%)
Other	17 (18.3%)
USD patients seen annually	
Less than 20	22 (23.7%)
Between 20 and 50	21 (22.6%)
More than 50	50 (53.8%)
Years in practice	
Still in training	7 (7.5%)
0-3	7 (7.5%)
3-5	6 (6.5%)
5-10	16 (17.2%)
More than 10	57 (61.3%)
Academic Affiliation	
Yes	82 (88.2%)
No	11 (11.8%)

### Use of 24-Hour Urine Testing

Sixty percent of respondents reported ordering 24-hour urine testing in all or more than half of their patients (Fig 2). The reported use of 24-hour urine testing varied by specialty (*P* < 0.001): 72% of nephrologists and 63% of urologists reported ordering 24-hour urine testing for all or more than half of their patients; in contrast, only 30% of providers other than nephrology and urology reported ordering 24-hour urine testing (Fig 2). Respondents were asked whether they would order 24-hour urine testing more if an educational tool for laboratory interpretation were available. Those who responded “yes” indicated an electronic health record-based tool would be the most effective teaching tool (94% strongly or somewhat agree) compared with an online course (71% strongly or somewhat agree) or website (80% strongly or somewhat agree) (Fig S1).

**Figure 2:**
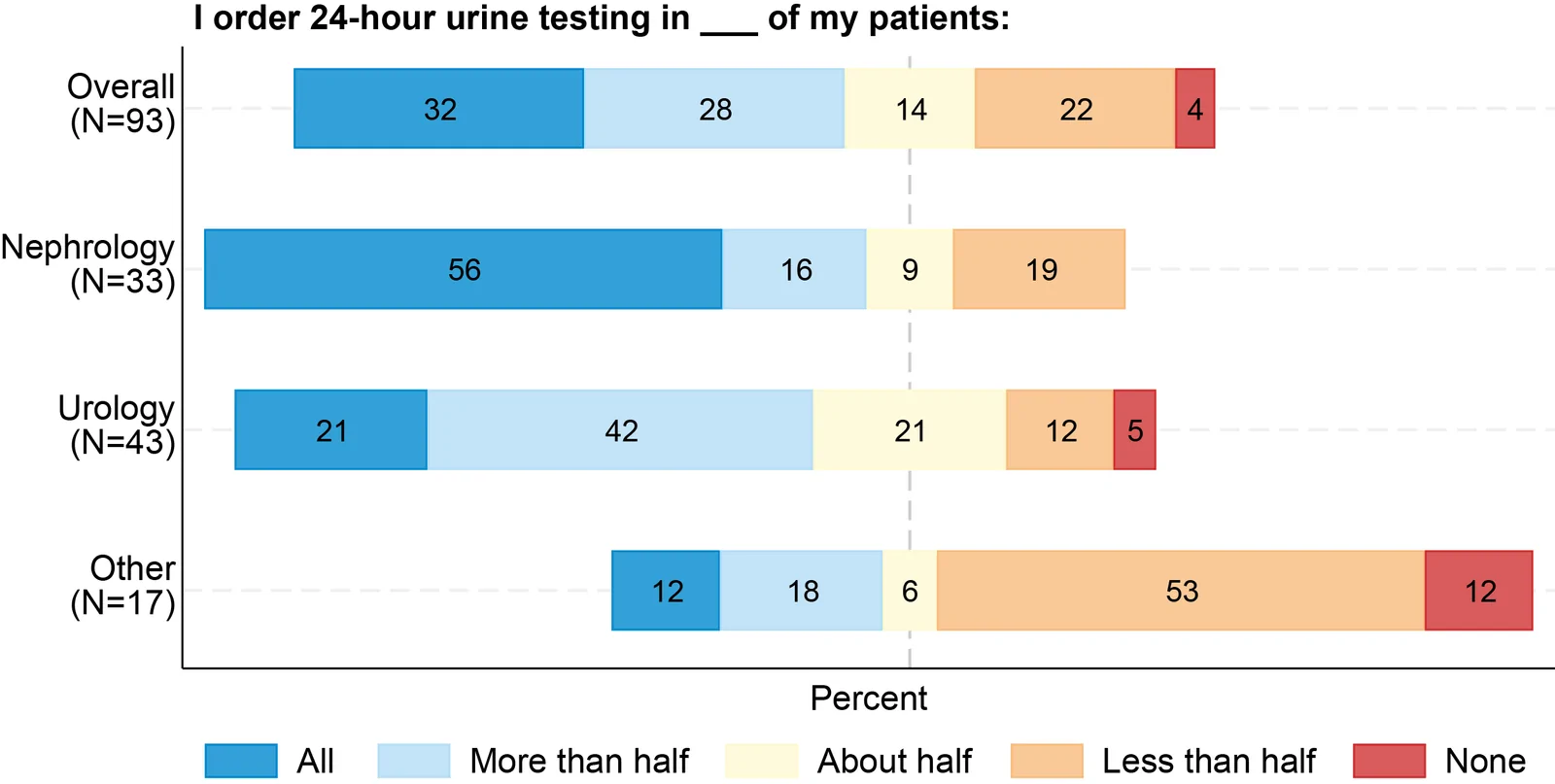
Twenty-four-hour urine testing for patients with urinary stone disease. Frequency by which respondents (n = 93) order 24-hour urine testing for patients with urinary stone disease. Frequency of 24-hour urine testing was also stratified by provider type: nephrology (n = 33), urology (n = 43), and other (n = 17).

Respondents were asked to rate their perception of barriers related to 24-hour urine testing (Fig 3). Most providers indicated that they view the test as necessary: for the statement “I don’t think it is necessary,” 65% strongly disagreed and 28% somewhat disagreed. Similarly, a majority did not view lack of knowledge as a barrier, as 84% strongly disagreed and 10% somewhat disagreed with the statement “I don’t know how to order it,” while 61% strongly disagreed and 20% somewhat disagreed with the statement “I don’t know how to interpret the results.” The most frequently cited barrier to 24-hour urine testing was patient burden, with 35% of respondents somewhat agreeing with the statement “It is too hard for my patients to collect.”

**Figure 3:**
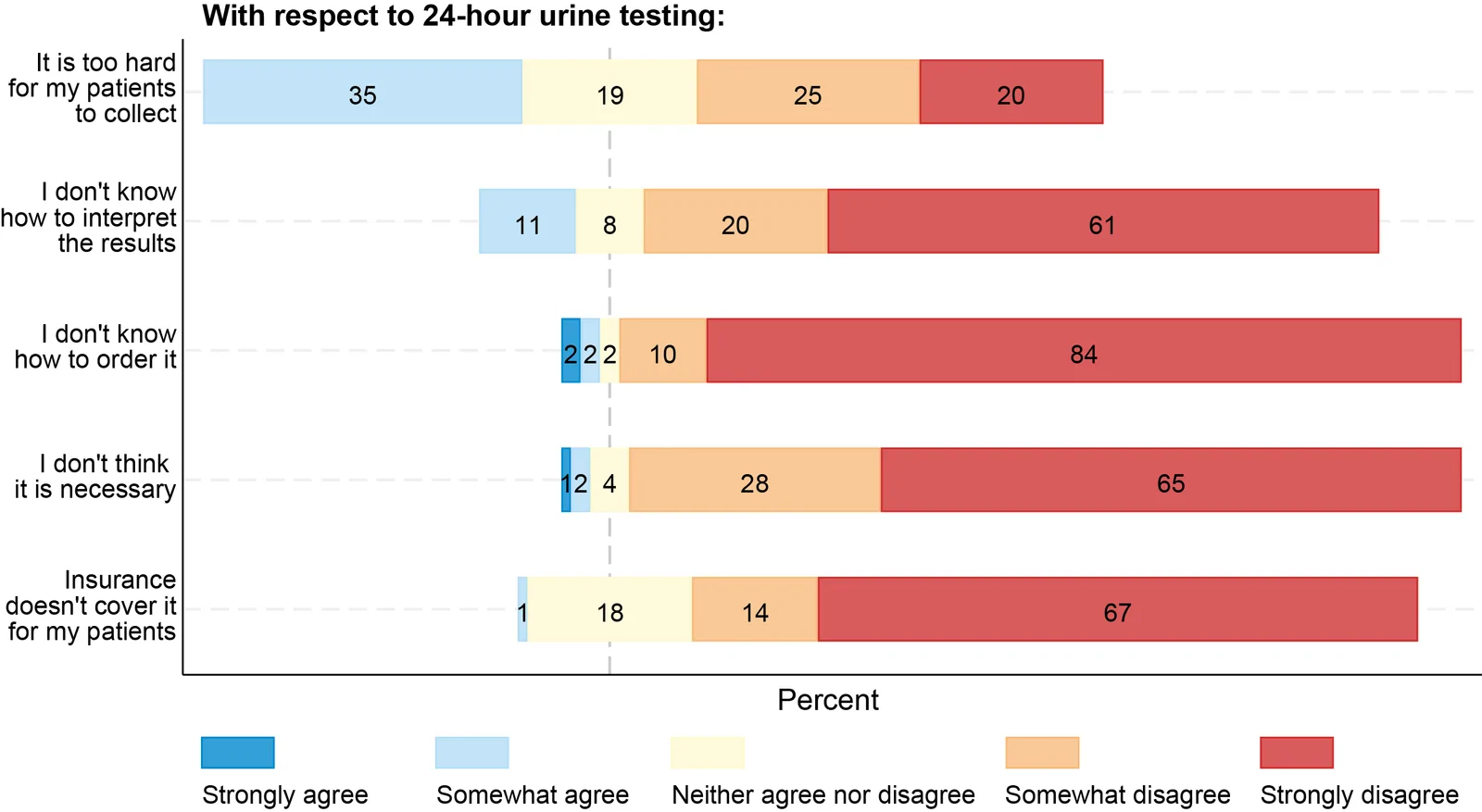
Perceived barriers to 24-hour urine testing by all respondents. Respondents were asked to rate their perception of barriers to 24-hour urine testing.

### Stone Prevention Measures

Among those who had access to 24-hour urine results, 77% (always or more than half of the time) indicated that they use the results to make dietary recommendations (Fig 4). Respondents were asked how frequently they make specific dietary recommendations for the most common stone type, calcium oxalate. Nearly all respondents (95%) reported advising patients to maintain high fluid intake “always” or “more than half” of their patients. Restriction of dietary sodium was also common, with 83% reporting that they recommended this advice to all or more than half of their patients. Fewer respondents reported routinely counseling patients to restrict dietary oxalate, with only 45% doing so to all or more than half of their patients (Fig 4).

**Figure 4:**
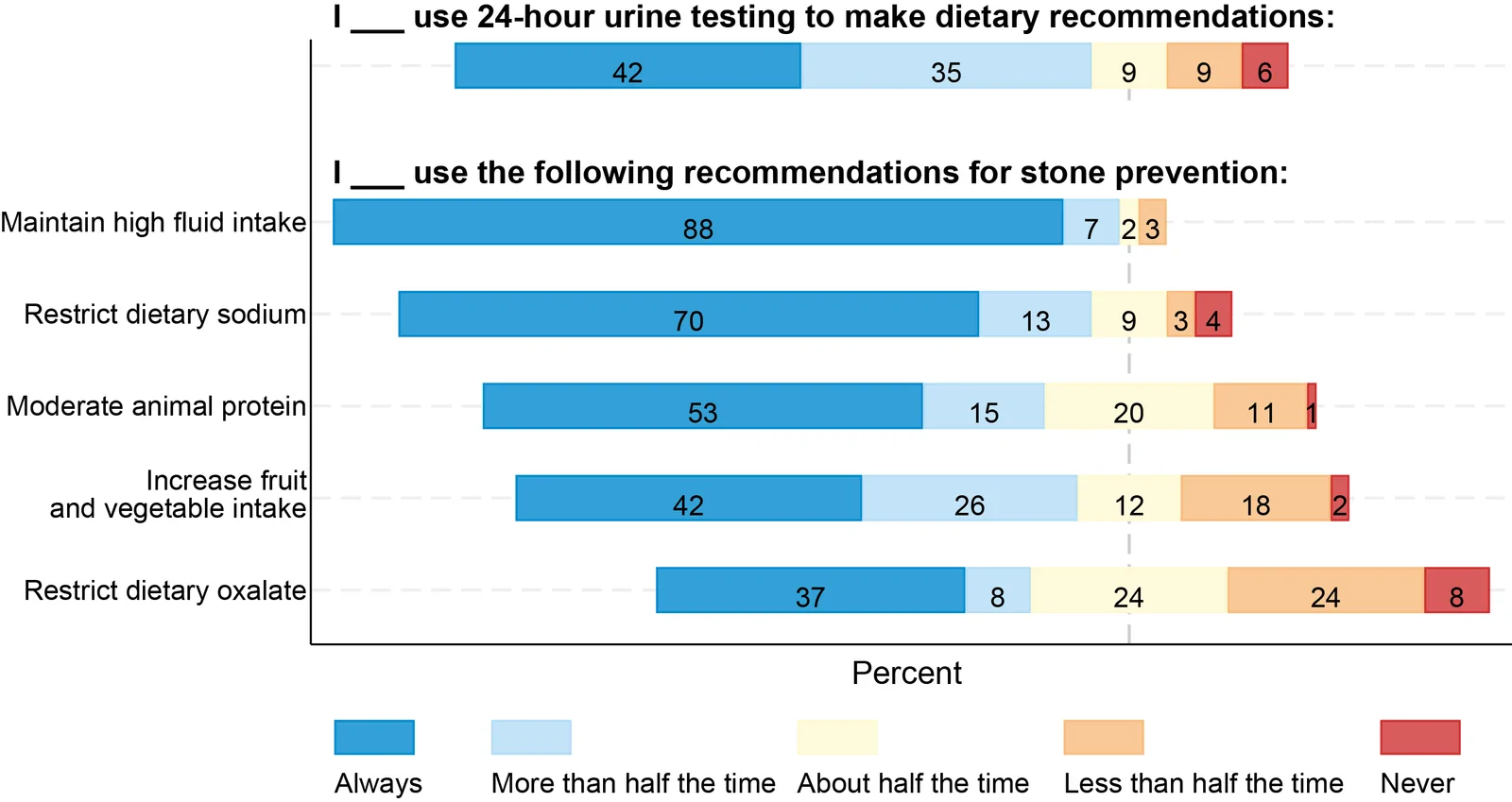
Twenty-four-hour urine testing for patients with urinary stone disease as a guide for dietary recommendations. Frequency by which respondents incorporate 24-hour urine testing in making dietary recommendations. Respondents were also asked about the types of dietary interventions they recommend to a typical calcium oxalate stone former.

Nearly all respondents (91%) reported using available 24-hour urine results to guide prescription decisions, with 63% doing so “always” and 28% doing so “more than half the time” (Fig 5A). When asked about which specific medications are prescribed, most providers reported prescribing alkali for hypocitraturia (always: 25%; more than half: 43%) and alkali for acidic urine (always: 20%; more than half: 32%). Thiazide or thiazide-type diuretics were prescribed by almost half of providers for hypercalciuria (always: 8%; more than half: 37%). Allopurinol for hyperuricosuria was prescribed the least, with 27% indicating that they had never prescribed the drug and 52% in fewer than half of their patients (Fig 5B). Patterns of pharmacologic therapy differed between nephrologists and urologists (*P* < 0.001). For alkali therapy in hypocitraturia, 80% of nephrologists reported prescribing alkali “always” or “more than half” of the time, compared with 70% of urologists. The greatest difference between nephrologists and urologists was observed for alkali therapy for acidic urine, with urologists reported prescribing alkali in 60% of cases (“always” or “more than half” of the time) compared with 45% of nephrologists. Patterns of pharmacologic therapy also differed between specialists (nephrologists and urologists) and generalists (other) (*P* < 0.001), with the greatest observed difference for alkali therapy for hypocitraturia, as 80% of nephrologists and 70% of urologists reported prescribing alkali (“always” or “more than half” of the time) compared with 30% of generalists (other).

**Figure 5:**
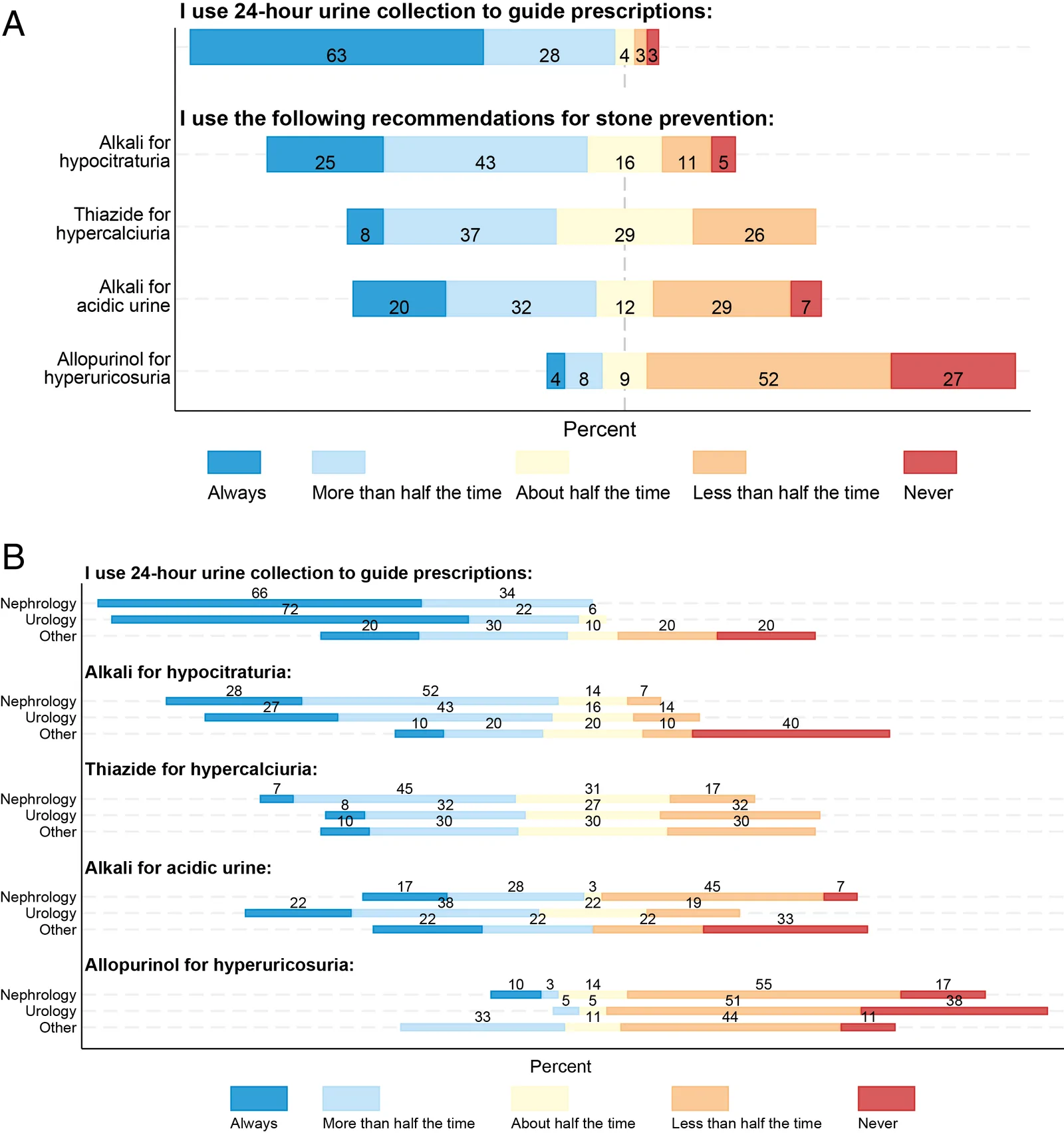
Twenty-four-hour urine testing for patients with urinary stone disease as a guide for prescriptions. (A) Frequency by which respondents use 24-hour urine testing in guiding prescription of medications for stone prevention. Respondents were also asked about the types of medications that they prescribe for specific 24-hour urine abnormalities. (B) Frequency by which respondents use 24-hour urine testing in guiding prescription of medications for stone prevention, stratified by provider type: nephrology (n = 33), urology (n = 43), and other (n = 17).

### Stone Surveillance

Respondents were asked whether they routinely order imaging studies for stone surveillance. Those who indicated “yes” (70%) were then asked to specify the types of imaging that they ordered, with options including renal ultrasound, computed tomography (CT) of the abdomen and pelvis without contrast, low-dose or ultra-low-dose CT abdomen and pelvis without contrast, or kidney, ureter, and bladder (KUB) radiograph. Most providers who use stone surveillance imaging (n = 63) ordered both ultrasound and CT (71%) (Fig 6). When examined by specialty, about one-third of nephrologists (32%) reported ordering CT alone, whereas over half of nephrologists (58%) reported ordering both ultrasound and CT. In contrast, only a small fraction of urologists (11%) indicated ordering CT alone, whereas most urologists (76%) indicated using both modalities (Fig 6). Although 83% of generalists (other) reported ordering both ultrasound and CT, 17% reported not ordering surveillance imaging at all. Respondents who do not order stone surveillance imaging were asked to select reasons why from a predefined list. The most frequently selected reason was reliance on patient symptoms to guide imaging decisions (80%), followed by concern that CT scanning increases radiation exposure to patients (25%) and concern that ultrasound and KUB have poor sensitivity to detect stones (10%).

**Figure 6:**
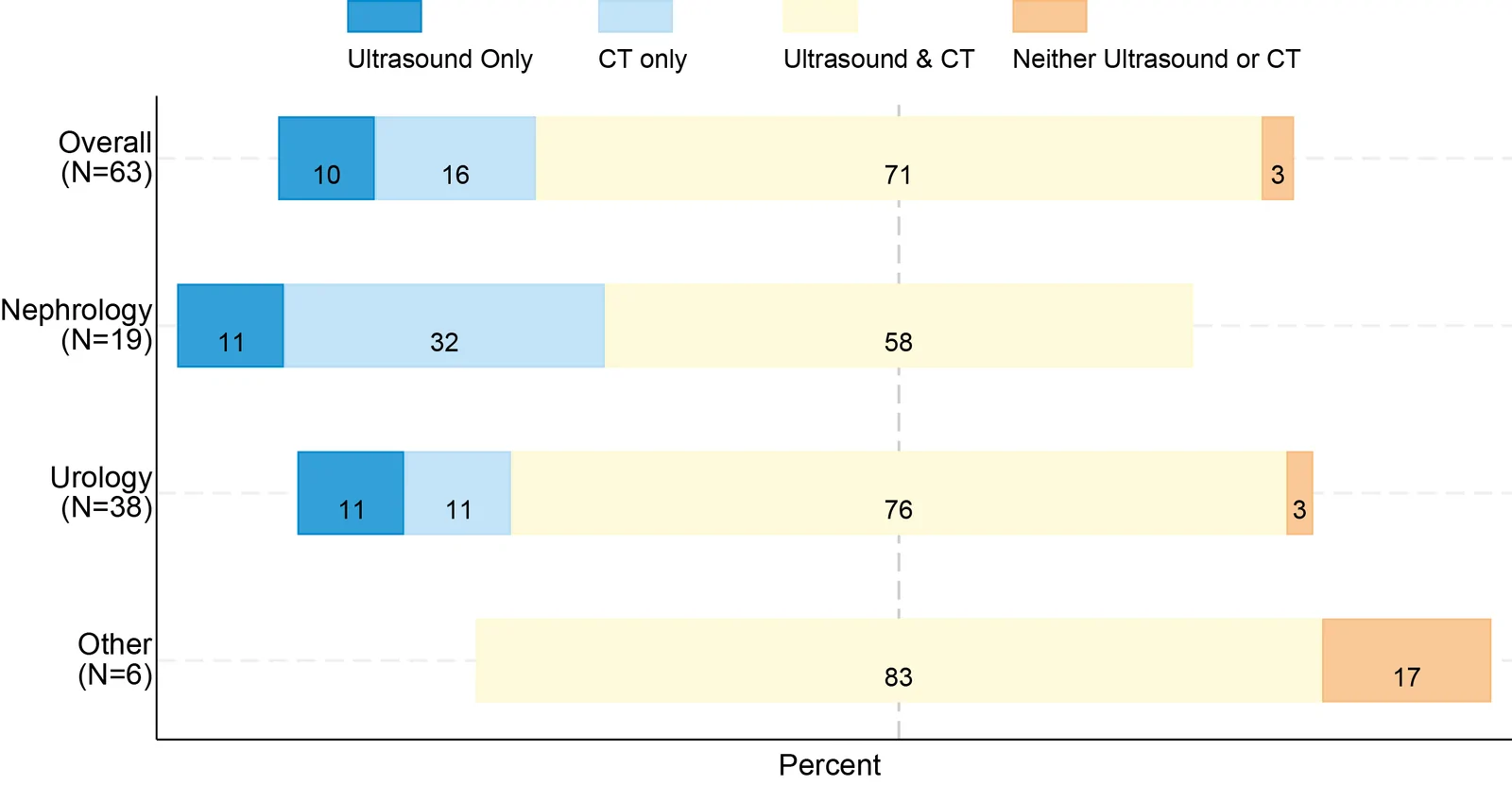
Stone surveillance imaging ordered by all respondents (overall) and by subspecialty. Participants were asked whether they routinely order imaging studies for stone surveillance. Out of 93 respondents, 65 responded that they did order imaging but 2 did not elaborate on the type of imaging. Neither ultrasound nor computed tomography category includes those who received kidney, ureter, and bladder radiographs.

## Discussion

We conducted a national survey of nephrology, urology, and primary care providers to identify current barriers to implementing stone prevention measures. Clinicians reported ordering 24-hour urine testing frequently and that they used test results to guide dietary and medical approaches for stone prevention. The main barrier to ordering a 24-hour urine test was the perception that a 24-hour urine sample is difficult for patients to collect. For stone surveillance imaging, most survey respondents used a combined approach of ultrasound and CT scan for evaluating patient stone burden. We also found subspecialties differ in how they approach stone prevention. Nephrologists were more likely to implement 24-hour urine testing and prescribe thiazide diuretics for hypercalciuria and alkali for hypocitraturia, whereas urologists were more likely to prescribe alkali for acidic urine. Overall, our survey identifies awareness, use, and opinions of health care providers regarding the evaluation and implementation of stone prevention measures, which can inform efforts to standardize clinical practice and fill gaps in the clinical evidence supporting the effectiveness of approaches to reduce stone recurrence.

Our survey found that health care providers, many of whom practice in stone specialty clinics, rely on 24-hour urine tests to guide dietary counseling and prescription decisions. The most common dietary recommendations were high fluid intake and low dietary sodium; in contrast, the least common dietary recommendation was low dietary oxalate. Indeed, observational and clinical trial data have demonstrated the clinical efficacy for fluid intake of more than 2 liters/day^11^^,^^12^ and dietary sodium < 100 mmol/day.^13^^,^^14^ Low dietary oxalate is also recommended for patients with hyperoxaluria, generally when 24-hour urine oxalate exceeds 25 mg/day,^15^ but we suggest that dietary oxalate restriction can be challenging for many patients. First, oxalate is found in many common foods and beverages, and so it can be difficult for patients to limit or avoid oxalate entirely. Second, patients with co-morbid disease such as hypertension or type 2 diabetes mellitus may need to balance other dietary recommendations simultaneously, which may make oxalate restriction more difficult. For example, the Mediterranean diet has been recommended for heart health, but this diet introduces more nuts for consumption, which may increase urinary oxalate excretion.^16^

The most common medication prescribed by survey respondents was alkali supplements, followed by thiazide diuretics, although almost half of health care providers still prescribed thiazide diuretics in all or more than half of patients with hypercalciuria. We suggest that recent findings from the NOSTONE trial, which demonstrated no apparent benefit in preventing stone recurrence after 3 years,^17^ have reduced the inclination to prescribe thiazides to patients who have hypercalciuria. In contrast, the relatively low side effect profile of alkali supplements, combined with having 2 indications for stone prevention, ie, hypocitraturia for calcium stones or acidic urine for uric acid stones, may encourage health care providers to prescribe alkali for stone prevention. Allopurinol, which can be prescribed for patients with hyperuricosuria and calcium oxalate stones, was prescribed rarely—only about 10% of survey respondents prescribed allopurinol for more than half or all their patients who met criteria for allopurinol treatment.

For stone surveillance imaging, most survey respondents used a combined approach of ultrasound and CT scan for evaluating patient stone burden. A quarter of respondents reported a concern that CT scanning exposes patients to ionizing radiation, while a tenth of respondents acknowledge that ultrasound or KUB has poor sensitivity for stone detection. The optimal strategy and clinical benefit of routine imaging for stone surveillance is uncertain, as current guidelines recommend that imaging be individualized based on patient risk, stone characteristics, and prior interventions.^2^^,^^18^ Most respondents (80%) reported ordering imaging only when patients are symptomatic, and this symptom-driven approach aligns with guideline recommendations to limit unnecessary radiation exposure.^18^^,^^19^

Finally, this survey identifies differences in how subspecialists evaluate and manage USD, especially regarding 24-hour urine testing and prescription of stone-related medications. Nephrologists were more likely to implement 24-hour urine testing and prescribe thiazide diuretics for hypercalciuria and alkali for hypocitraturia, whereas urologists were more likely to prescribe alkali for acidic urine. These findings suggest that differences in clinical training or clinical focus (eg, prescribing treatments to alkalinize the urine to avoid surgery for uric acid stones) may shape provider-specific approaches to stone prevention. Regarding stone surveillance imaging, both nephrologists and urologists favored a combined approach of ultrasound and CT scanning, although nephrologists were more likely to use CT alone. We suggest that urologists may be more aware of ultrasound-first strategies that can detect or monitor suspected USD without exposing patients to radiation.

Results from this survey are consistent with prior studies examining practice patterns in evaluation and management of USD. In one survey that included both patients and providers, patients cited difficulty collecting 24-hour urine samples and lack of clear instructions as major barriers to implementing 24-hour urine testing.^20^ A second survey, which included 305 urologists across 57 different countries, also found that low rates of 24-hour urine testing were attributable to patient-related factors.^21^ Our survey indicates that the reasons for the low prevalence of 24-hour urine testing in clinical practice are not attributable to lack of insurance reimbursement or difficulty in interpreting results. Instead, respondents indicated that they consider the test necessary and felt confident in ordering and interpreting results. It is possible that health care providers in our survey, many of whom practice in stone specialty clinics, rely on 24-hour urine tests because they have the clinical expertise to incorporate urine chemistry data for guiding dietary counseling and prescription decisions.

The strengths of this study include the national distribution of our survey, the inclusion of multiple provider types, and the representation of providers who care for a high volume of patients with USD, with over half reporting that they manage more than 50 patients with USD annually. However, we note the following limitations. First, the sample may not be representative of all nephrologists and urologists practicing in the United States. We recruited respondents through national conferences and educational sessions focused on USD, which likely attracted respondents with more interest or expertise in stone prevention or those affiliated with academic institutions. The higher proportion of respondents who are familiar with 24-hour urine testing and stone prevention measures may differ from a survey with a more general provider population in community and private practice. Future studies should aim to include a more representative sample of these providers to capture real-world stone care across these more diverse settings.

In summary, this national survey demonstrates that most nephrologists and urologists view 24-hour urine testing as an essential component of USD management. However, patient-level barriers, specifically the perceived burden of 24-hour urine collection, remain a significant obstacle to broader implementation. When 24-hour urine testing is performed, results are widely used to inform dietary and pharmacologic interventions. Variation in stone prevention strategies is apparent between nephrologists and urologists in their approach to evaluating and monitoring the burden of USD. Efforts to standardize care across subspecialties and improve patient education and engagement may meaningfully increase adherence to guideline-recommended preventive care and reduce the overall burden of recurrent USD.
