# Supplementary material for: A National Survey of Provider Practice Patterns for Evaluation and Management of Patients With Urinary Stone Disease
Source: Kidney Med. 2026 Sep 9;8(12):101520. doi: 10.1016/j.xkme.2026.101520 (PMC13756155; doi:10.1016/j.xkme.2026.101520)
Supplement: Supplementary file 1 [file mmc1.pdf]

Supplementary Material

Table S1. American Urological Association Guidelines for the Medical Management of Urinary Stone Disease

**Evaluation**	
Clinicians should perform additional metabolic testing in high-risk or interested first-time stone formers and recurrent stone formers.	Standard; Evidence Strength: Grade B*
Metabolic testing should consist of one or two 24-hour urine collections obtained on a random diet and analyzed at minimum for total volume, pH, calcium, oxalate, uric acid, citrate, sodium, potassium and creatinine.	Expert Opinion*
**Diet Therapies**	
Clinicians should recommend to all stone formers a fluid intake that will achieve a urine volume of at least 2.5 liters daily.	Standard; Evidence Strength: Grade B
Clinicians should counsel patients with calcium stones and relatively high urinary calcium to limit sodium intake and consume 1,000-1,200 mg per day of dietary calcium.	Standard; Evidence Strength Grade: B
Clinicians should encourage patients with calcium stones and relatively low urinary citrate to increase their intake of fruits and vegetables and limit non-dairy animal protein.	Expert Opinion
Clinicians should counsel patients with calcium oxalate stones and relatively high urinary oxalate to limit intake of oxalate-rich foods and maintain normal calcium consumption.	Expert Opinion
**Pharmacologic Therapies**	
Clinicians should offer potassium citrate therapy to patients with recurrent calcium stones and low or relatively low urinary citrate.	Standard; Evidence Strength Grade B
Clinicians should offer thiazide diuretics to patients with high or relatively high urine calcium and recurrent calcium stones.	Standard; Evidence Strength Grade B
Clinicians should offer potassium citrate to patients with uric acid and cystine stones to raise urinary pH to an optimal level.	Expert Opinion
Clinicians should offer allopurinol to patients with recurrent calcium oxalate stones who have hyperuricosuria and normal urinary calcium.	Standard; Evidence Strength Grade B
**Follow-up**	
Clinicians should periodically obtain follow-up imaging studies to assess for stone growth or new stone formation based on stone activity (plain abdominal imaging, renal ultrasonography or low dose computed tomography.)	Expert Opinion

*When sufficient evidence existed, the body of evidence for a particular clinical action was assigned a strength rating of A (high), B (moderate) or C (low). In the absence of sufficient evidence, additional information is provided as Clinical Principles and Expert Opinions.

Figure S1. Reported usefulness of various educational tools for interpreting 24-hour urine testing. Participants were asked whether they would order 24-hour urine testing more if an educational tool for laboratory interpretation were available. Out of 93 responders, 51 responded not using 24-hour urine testing and 11 did not respond. Percents calculated among the 31 responders who responded "yes".

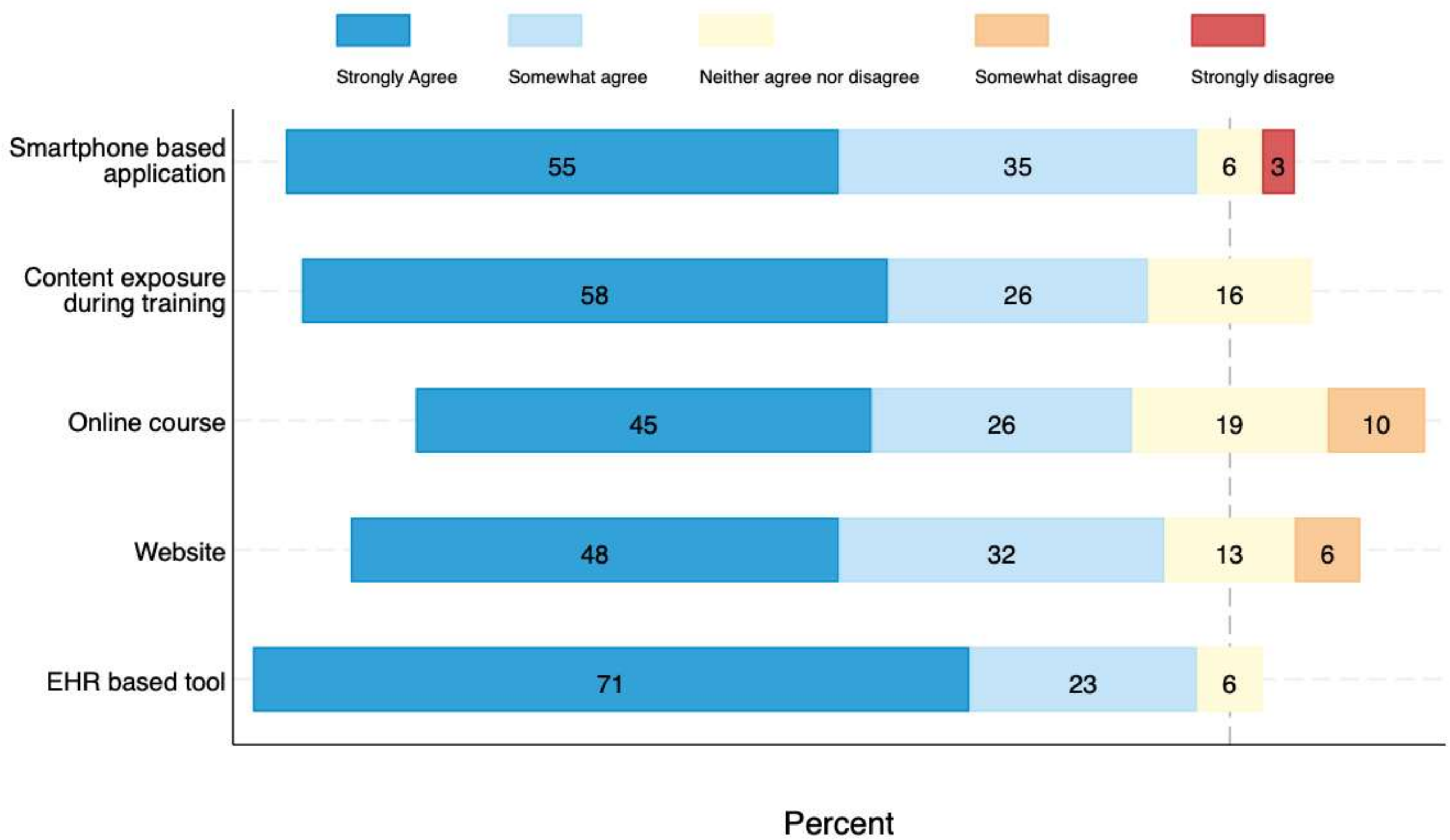

Table S2. Stone Care Guidelines Survey

Start of Block: Screening question

Q1 Which of the following best describes your current position?

- Nurse Practitioner
- Urologist
- Physician
- Pharmacist
- Physician-assistant
- Social worker
- Dietician
- None of the above
- Other ____

Q2 Do you work in the United States?

- Yes
- No

Q3 How many patients with kidney or ureteral stones do you see in a year?

- None
- Less than 20 patients
- Between 20 and 50 patients
- More than 50 patients

Start of Block: Background on participant

Q4 Do you work at the Veterans Health Administration?

◯ Yes

◯ No

Q5 Which of the following best describes the setting you practice in? (Select all that apply.)

▢ Ambulatory Clinic (outpatient)

▢ Emergency Department

▢ Hospital (inpatient)

▢ Other ____

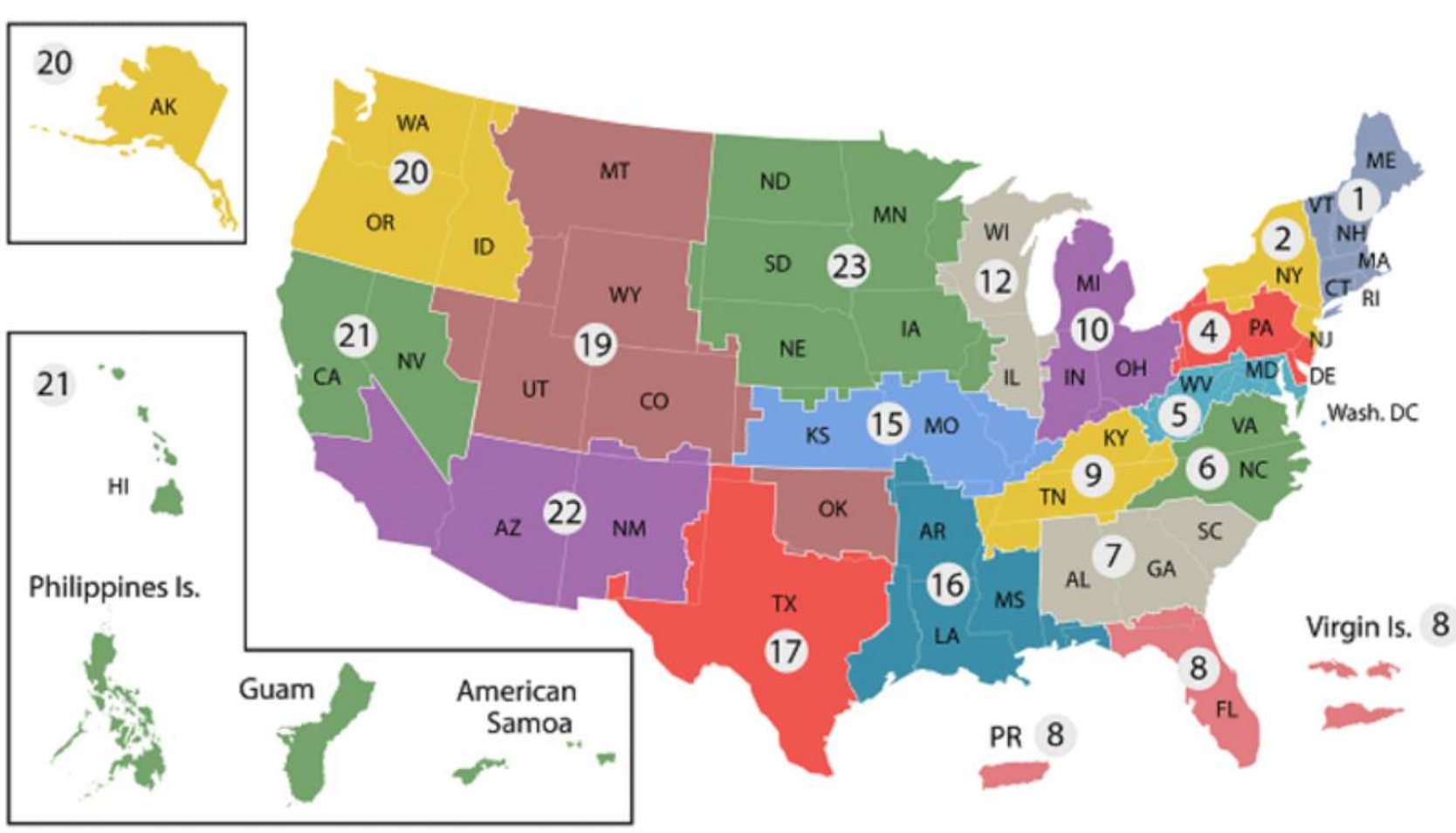

Q6 Enter the number of the region of where you practice.

____

Q7 Is your site affiliated with an academic institution?

- ◯ Yes
- ◯ No

Q8 My area or specialty would be best described as

- ◯ Primary care
- ◯ Hospitalist medicine
- ◯ Nephrology
- ◯ Urology
- ◯ Endocrinology
- ◯ Other ____

Q9 How many years have you been in practice?

- ◯ Still in training
- ◯ 0-3 years
- ◯ 3-5 years
- ◯ 5-10 years
- ◯ Over 10 years

Start of Block: 24-hour urine testing

Q10 I order 24-hour urine testing in

- ○ all my patients
- ○ more than half of my patients
- ○ about half of my patients
- ○ less than half of my patients
- ○ none of my patients

Q11 With regard to 24-hour urine testing...

	Strongly Agree	Somewhat agree	Neither agree nor disagree	Somewhat disagree	Strongly disagree
I don't think it is necessary	○	○	○	○	○
I don't know how to order it	○	○	○	○	○
Insurance doesn't cover it for my patients	○	○	○	○	○
It is too hard for my patients to collect	○	○	○	○	○
I don't know how to interpret the results	○	○	○	○	○

Start of Block: Specific Recommendations

Q12 How often do you rely on information from the following tests (if available) to prevent future stones?

	Always	Most of the time	About half the time	Sometimes	Never
Serum basic metabolic panel	○	○	○	○	○
Serum parathyroid hormone	○	○	○	○	○
Stone analysis	○	○	○	○	○

Q13 Do you make dietary recommendations for stone prevention?

○ Yes

○ No

Q14 How often do you use the 24-hour urine collection (if available) to make dietary recommendations?

○ Always

○ More than half the time

○ About half the time

○ Less than half the time

○ Never

Q15 For your typical calcium oxalate stone former, do you make the following dietary recommendations for stone prevention?

	Always	Less than half the time	About half the time	More than half the itme	Never
Maintain high fluid intake	○	○	○	○	○
Restrict dietary sodium	○	○	○	○	○
Restrict dietary oxalate	○	○	○	○	○
Increase fruit and vegetable intake	○	○	○	○	○
Moderate animal protein	○	○	○	○	○

Q16 The reason I don't make dietary recommendations include (click all that apply)

- ☐ unaware of dietary recommendations
- ☐ not enough time to review diet
- ☐ no dietician support
- ☐ lack of evidence in support of dietary change

Q17 Do you prescribe medications for stone prevention?

- ○ Yes
- ○ No

Q18 How often do you use the 24-hour urine collection (if available) to guide prescriptions?

- Always
- More than half the time
- About half the time
- Less than half the time
- Never

Q19 How often do you prescribe the following?

	Always	More than half the time	About half the time	Less than half the time	Never
Thiazide for hypercalciuria	○	○	○	○	○
Alkali for hypocitraturia	○	○	○	○	○
Alkali for acidic urine	○	○	○	○	○
Allopurinol for hyperuricosuria	○	○	○	○	○

Q20 The reason I don't prescribe medications includes (click all that apply)

- ☐ unaware of which medications to prescribe
- ☐ lack of evidence that medications work
- ☐ lower clinical priority
- ☐ too many side effects

Q21 Do you order imaging for stone surveillance?

- Yes
- No

Q22 What type of imaging study do you order? (click all that apply)

- Renal Ultrasound
- KUB
- CT Abdomen and Pelvis without contrast
- CT Abdomen Pelvis without contrast (low dose or ultra-low dose)

Q23 The reason I don't order imaging for stone surveillance includes (click all that apply)

- CT increases radiation exposures
- Imaging doesn't change management
- Ultrasound or KUB has poor sensitivity
- Patient symptoms prompt imaging

Start of Block: Educational materials

Q24 I would use 24-hour urine testing more if I had an education tool for lab interpretation

- Yes
- No

Q25 The following format for an education tool would be helpful

	Strongly Agree	Somewhat agree	Neither agree nor disagree	Somewhat disagree	Strongly disagree
EHR based tool	○	○	○	○	○
Website	○	○	○	○	○
Online course	○	○	○	○	○
Content exposure during training	○	○	○	○	○
Smartphone based application	○	○	○	○	○
